# Measuring Success: Key Findings From the OCEBHA Annual Conference Survey

**DOI:** 10.1093/geroni/igaf122.1980

**Published:** 2025-12-31

**Authors:** Julia Unsworth, Lindsey Smith

**Affiliations:** OHSU-PSU School of Public Health, Portland, Oregon, United States; Oregon Health & Science University, Portland, Oregon, United States

## Abstract

In assessing training needs, a strong demand was identified for specialized behavioral health training for professionals working with older adults, as well as targeted aging-related training for those working in the behavioral health field. To address this need, OCEBHA held its first statewide conference in 2024, selling out and drawing over 150 professionals. The two-day conference covered topics related to integrative behavioral health care for older adults. The OCEBHA evaluation team collected surveys (60% response rate) and conducted onsite interviews. Most attendees worked in direct care or social work, primarily within behavioral health or aging and disability services. Nearly all survey respondents (92%) were likely to apply conference knowledge in their work and 96% planned to share insights with colleagues. The majority of respondents gained valuable knowledge on substance abuse in older adults (88%), challenges for older adults with SMI (89%), and geriatric care (87%). All respondents (100%) reported learning at least a little about these core conference learning objectives. Attendees appreciated the conference’s focus on behavioral health and aging, an often neglected issue highly relevant to the challenges of many attendees’ work. Respondents valued the event’s focus on diverse topics, expert speakers, networking opportunities, and the overall organization and engagement of the event. Overall, the conference team exceeded their goal of 100 attendees but aims to expand engagement across Oregon and beyond.

